# Fecal Microbial Communities in a Large Representative Cohort of California Dairy Cows

**DOI:** 10.3389/fmicb.2019.01093

**Published:** 2019-05-16

**Authors:** Jill V. Hagey, Srijak Bhatnagar, Jennifer M. Heguy, Betsy M. Karle, Patricia L. Price, Deanne Meyer, Elizabeth A. Maga

**Affiliations:** ^1^Department of Animal Science, University of California, Davis, Davis, CA, United States; ^2^Geomicrobiology Group, Department of Biological Sciences, University of Calgary, Calgary, AB, Canada; ^3^Division of Agriculture and Natural Resources, University of California Cooperative Extension, Hayward, CA, United States

**Keywords:** rumen microbial analysis, dairy cattle, California dairies, 16S/18S ribosomal RNA gene analysis, rumen, microbiome

## Abstract

Improved sequencing and analytical techniques allow for better resolution of microbial communities; however, the agriculture field lacks an updated analysis surveying the fecal microbial populations of dairy cattle in California. This study is a large-scale survey to determine the composition of the bacterial community present in the feces of lactating dairy cattle on commercial dairy operations. For the study, 10 dairy farms across northern and central California representing a variety of feeding and management systems were enrolled. The farms represented three typical housing types including five freestall, two drylot and three pasture-based management systems. Fresh feces were collected from 15 randomly selected cows on each farm and analyzed using 16S rRNA gene amplicon sequencing. This study found that housing type, individual farm, and dietary components significantly affected the alpha diversity of the fecal microbiota. While only one Operational Taxonomic Unit (OTU) was common among all the sampled individuals, 15 bacterial families and 27 genera were shared among 95% of samples. The ratio of the families *Coriobacteriaceae* to *Bifidobacteriaceae* was significantly different between housing types and farms with pasture fed animals having a higher relative abundance of *Coriobacteriaceae*. A majority of samples were positive for at least one OTU assigned to *Enterobacteriaceae* and 31% of samples contained OTUs assigned to *Campylobacter*. However, the relative abundance of both taxa was <0.1%. The microbial composition displays individual farm specific signatures, but housing type plays a role. These data provide insights into the composition of the core fecal microbiota of commercial dairy cows in California and will further generate hypotheses for strategies to manipulate the microbiome of cattle.

## Introduction

The bovine gastrointestinal microbiota is comprised of protozoa, archaea, bacteria, and fungi representing a complex ecosystem that digests feed providing vitamins, volatile fatty acids and other nutrients for their hosts. While culture-dependent methods to determine rumen microbial composition started with vigor in the 1940’s, current accessibility of modern sequencing technologies that leverage culture independent methods have positioned the microbial ecology of cattle for its renaissance (Bryant, 1959; Hungate et al., 1964). Most molecular efforts of microbial community identification in cattle have focused on the rumen microbiota, with less attention paid to determining the communities of fecal microbiota (Dowd et al., 2008; Shanks et al., 2011; Tang et al., 2017). Elucidating the fecal microbiota of dairy cattle has implications for mitigating environmental impacts of pollution as well as improving manure management.

Previous studies, using 16S rRNA gene sequencing-based approaches, to investigate the effects of a variety of feeds on the fecal microbiota of cattle provided valuable initial insights (Dowd et al., 2008; Callaway et al., 2010; Rice et al., 2012; Li et al., 2017). Now due to the low cost of high-throughput sequencing, an updated and comprehensive view of the fecal microbiome with larger samples sizes for robust analysis can be achieved that will improve on earlier studies of the bovine fecal microbiome (Durso et al., 2010; Rudi et al., 2012; Kim et al., 2014). In addition, the significant advances in sequence data analysis provides an opportunity for more refined insights into this ecosystem (McMurdie and Holmes, 2014).

As of 2017, California is home to 1.73 million milk cows and roughly an equal number of replacement heifers (California Department of Food and Agriculture [CDFA], 2018). During lactation the average adult cow produces roughly 65 kg of manure a day, which presents a considerable waste management challenge (American Society of Aricultural and Biological Engineers [ASABE], 2005). Manure collection, treatment, storage and utilization are typically determined by the farm’s housing type. Manure from drylots is removed in solid form in spring and fall (Meyer et al., 2011). Freestall dairies in California are open-sided barn structures with concrete flooring and elevated platforms with bedded individual cattle resting spaces. These dairies predominantly collect manure as a liquid by scraping or flushing lanes one to four times a day. The waste stream usually undergoes solid-liquid separation before being stored in open atmosphere anaerobic storage structures. Separated solids are often dried and used for animal bedding. Additionally, solids and liquids are applied to land as a fertilizer for crops eaten by livestock, as the nitrogen in urine and feces makes manure a potent fertilizer. While use of manure reduces the need for chemical fertilizers, correct management remains important to avoid pollution.

Fecal nitrogen is excreted into the environment in the form of ammonium (NH^+^_4_), urea and organic nitrogen. The metabolic activity of microorganisms then convert these to other forms of nitrogen including ammonia (NH_3_), dinitrogen (N_2_), nitrous oxide (NO), nitric oxide (N_2_O), and nitrate (NO^–^_3_), which can potentially impact air, soil and water quality (Richardson et al., 2009; Tamminga, 2017). In recent years, increased nitrate concentration in the ground water of the Central Valley has increased pressure on California’s dairies to reduce their contributions of this pollutant (Harter and Lund, 2012). Furthermore, an increased push for renewable energy in California has fostered interest in using manure for anaerobic digesters to generate biogas. Information on a common fecal microbial community and how variables on farms change these communities will contribute to understanding the fertilization and energy potential of manure. Therefore, surveying the communities entering the manure storage lagoons is necessary to understand the role they play in nitrogen cycling.

In addition to the role fecal microbes play in the nitrogen cycle, dairy cattle are reservoirs for zoonotic bacterial pathogens such as *Escherichia coli* O157:H7, *Salmonella* spp. (Abu Aboud et al., 2016) and *Campylobacter jejuni* (USDA, 2011). Both *E. coli* and *Salmonella* can survive in soil when introduced via application of slurry – a mixture of urine and feces – or manure to crops (Semenov et al., 2009). This poses a public health risk if these waste borne pathogens percolate into the water supply. Although extensive attention has been paid to monitoring pathogens, a broader approach exploring shedding of *Enterobacteriaceae* is valuable as commensals in this family can both benefit the host through colonization resistance and be detrimental to public health by harboring antibiotic resistant genes (Bailey et al., 2010; Maltby et al., 2013). Surveying fecal communities will provide understanding of pathogen shedding in context with the rest of the microbiota.

Here we investigated the fecal microbiota of 150 dairy cows from 10 commercial farms across California using 16S rRNA gene amplicon sequencing. The goal of this study was to characterize the fecal bacterial community in several representative cohorts of dairy cattle and determine variation seen across housing types and farms. A common core microbiome defined at the species level in cattle is likely to be hampered by heterogeneous genotypes, environments and diets. In a previous study, pyrosequencing of rumen microbiota of dairy cows found the communities were phylogenetically related, but at the Operational Taxonomic Unit (OTU) and phylum level there was a high amount of variability among individuals in the study (Jami and Mizrahi, 2012). Similarly, high variability in microbial species abundance from animal to animal was seen in fecal samples (Durso et al., 2010). Therefore, we hypothesized there will be high variability at the species level, but a “core” fecal microbiota can be defined at a higher taxonomic level. This study presents an analysis of Illumina sequenced 16S rRNA gene amplicons to survey the fecal microbiome of commercial dairy cattle across California. Our data contributes to elucidating bacterial community structure and identification of families of interest for further studies into their functional roles.

## Materials and Methods

### Dairy Farms and Sample Collection

Commercial dairy farms enrolled in the project were in the two major regions for dairy farming in California, the Central Valley (*n* = 8) and North Coast (*n* = 2). Farms were privately owned and permissions to collect fecal samples were given by the owners of the farms. These farms ranged in size from near 100 to over 1,800 lactating cows. The predominant breed was Holstein (dairies 1–7, 10), while dairies 8–9 had all or mostly Jersey cattle. Farms varied in their housing type, with cattle on farms 1, 2, 3, 6, and 10 housed in freestalls, while those on farms 4 and 5 were on drylots. Pasture-based farms were 7, 8, and 9, which provided freestalls (farms 7 and 8) and loafing shed housing (farm 9) when weather prohibited pasture use. A pasture dairy, farm 9, offered supplemental forage (hay or alfalfa pellets) when pasture production was insufficient. Farms 7 and 8 also offered supplemental feed including grain. For the purpose of analyses, these facilities are identified as pasture-based dairies because at least 30% of the animals’ dry matter intake was consumed as fresh pasture per the National Organic Program Pasture Rule (United States Department of Agriculture et al., 2011). All cattle were fed to the National Research Council’s (NRC) standards to meet nutritional requirements for dairy cattle. Farms 1 through 7 and 10 fed total mixed rations (TMR) with heterogenous ingredients for all or part of the diet (Supplementary Table S1). Freestall dairies provided an open-sided barn with individual stalls for animals to lay in with access to an earthen lot for exercise. Animals in drylot dairies had a concrete feed apron for feeding and otherwise resided on earthen lots with shade structures. Groups of cattle were identified on each farm by stage of lactation. Within group, animals were selected randomly to obtain enough first lactation and animals in greater lactations. Fecal samples were collected mid-air from 15 randomly selected healthy cows by holding a clean labeled sampling container behind the defecating animals. Sealed sample containers were placed in ice and transported at 4°C to UC Davis arriving within 36 h of collection. All samples were frozen immediately upon arrival in the laboratory and stored at −20°C until analyzed.

### DNA Extraction and 16S rRNA Gene Sequencing

DNA was extracted from 150 mg of a homogenous fecal sample using the ZR Fecal DNA MiniPrep kit (Zymo Research, Irvine, CA, United States) with the included standard protocol. The extracted genomic DNA was quantified via NanoDrop and stored at −20°C. The V4 region of the 16S rRNA gene in the DNA was amplified using barcoded broad range primers. The forward primer, F515, contained a unique 8 bp barcode (X) linker region (GT), and an Illumina adapter (5′-XXXXXX**GT**GTGCCAGCMGCCGCGGTAA-3′). The reverse primer used was R806 (5′-GGACTACHVGGGTWTCTAAT-3′) (Caporaso et al., 2012). Individual indices for each library can be found in Supplementary Table S2. The amplification was carried out using GoTaq^®^Green Master Mix (Promega, Madison, WI, United States) as previously described (Mon et al., 2015). Briefly, each sample was PCR amplified in triplicate and the triplicates were combined and visualized on an agarose gel. These verified index libraries were pooled then purified using the QIAquick PCR Purification Kit (QIAGEN, Hilden, Germany). Nuclease free water was amplified with samples from each farm and sequenced as controls for kit contamination. The pooled samples were sequenced at the DNA Technologies Core of University of California, Davis Genome Center on the Illumina MiSeq PE250 platform.

### Amplicon Library Processing

Raw paired end reads were joined using FLASH2 v.c41a82e (Magoc and Salzberg, 2011). Merged reads were trimmed to remove adaptors and low quality bases (*Q* < 25) with Trimmomatic v.033 (Bolger et al., 2014). Any read smaller than 200 bp was discarded. The libraries were then screened for phiX contamination using FastQ Screen v.0.5.2 with the phiX174 reference genome (Genbank Acc# NC_001422.1). Reads longer than 302 bp were determined to be eukaryotic origin (host and protist) by BLAST and hence removed from the data set using screen.seqs function in mothur v.1.37.0 (Schloss et al., 2009). Sequences were demultiplexed in QIIME v.1.9.1 and the chimeras were identified and removed using the usearch61 function of QIIME (Caporaso et al., 2010b; Edgar, 2010). The sequences were then assigned to an OTU using *de novo* clustering. Subsequently, the OTUs were aligned with PyNAST and assigned taxonomy using RDP classifier with Greengenes reference database v.13_8. A phylogenetic tree of OTUs was made using FastTree with default options (Wang et al., 2007; Price et al., 2009; Caporaso et al., 2010a). All OTUs classified as mitochondria or chloroplast were removed. The OTU table and tree produced in QIIME were imported into the R package Phyloseq (McMurdie and Holmes, 2013). To reduce erroneous reads due to PCR errors and to adjust for sparsity of the OTU table for a more powerful analysis, only OTUs present in at least 3 samples with 3 or more reads were retained for downstream analyses.

### Microbial Community Analyses

In the DESeq2 R package, the OTU table was transformed using variance stabilizing transformation to account for differences in library size and significant log2 fold differences between housing types were determined (Love et al., 2014). Because the transformation resulted in negative log values for OTUs with a count close to zero, these negative values were changed to zero for ordination and for intuitive visualization of graphs. In QIIME, alpha diversity was calculated as Faith’s Phylogenetic Diversity (PD). Beta diversity was calculated by using unweighted UniFrac distances and graphed by Principal Component Analysis (PCoA) in the Phyloseq package. Sequence coverage was calculated with Zhang-Huang’s metric in the entropart R package (Marcon and Hérault, 2015), which uses the whole distribution to determine sequence coverage (Chao et al., 1988; Zhang and Huang, 2007). To elucidate associations between metadata and the microbial community abundance, a boosted additive general linear model was implemented with MaAsLin in R (Morgan et al., 2012). To further investigate taxonomy of sequences assigned to families containing zoonotic pathogens, a reference tree was built with FastTree (Price et al., 2010) from near full-length 16S rRNA gene sequences of various *E. coli* strains, relevant *Salmonella* serovars and *Campylobacter* species retrieved from the RDP database. Additionally, sequences that returned the max alignment score when query sequences were blasted against NCBI 16S ribosomal RNA sequence database were included in the reference tree. Query sequences were placed on this reference tree using Pplacer v. 1.1.alpha18 with default options (Matsen et al., 2010).

### Data Availability

Custom scripts for sequence processing, analysis and the full tree identifying pathogens can be found can be found at https://github.com/jvhagey/CDRF-CA_Dairy_Fecal_Microbiome/. Raw sequencing files are available through the Sequence Read Archive under the study accession number SRP115649.

### Statistics

Differential OTU abundance testing for housing types was carried out in DESeq2 using housing type as a covariate and Benjamini–Hochberg (BH) adjustment for multiple tests. An adjusted *p*-value ≤ 0.01 was considered significant. To test for significant differences between housing types while controlling for other factors a subset of farms (2–3, 7–8, and 10) that had complete records of metadata were analyze using a nested design with MaAsLin in R (Morgan et al., 2012). MaAsLin uses a general linear model with boosting to identify significant differences in microbial abundances associated with metadata. This method captures effects of a parameter of interest while deconfounding the effects of other metadata. Dietary variables in the model included hay or pasture for forage as well as the presence or absence of corn, silage, and pellets. Additionally, animal characteristics such as age, parity and stage of lactation were also included in the model. Both dietary variables and animal characteristics were entered as main effects in the model. As each farm only had one breed on site this source of variance could not be uncoupled from the variable farm and thus was not included in the model. A false discovery rate (FDR) corrected *p*-value, *q*-value, of ≤0.05 was considered significant. Significance of clustering was determined by PERMANOVA in the Vegan R package (Dixon, 2003). When the data met assumptions, the difference in housing types was tested using ANOVA, else the Kruskal–Wallis rank sum test was employed. For these tests, a *p*-value ≤ 0.05 was considered significant.

## Results

### Herd Statistics

The average number of lactations for enrolled animals was 2, with 41.6% first lactation heifers and 30.4% cows in lactations two or greater (Supplementary Table S2). Twelve and a half percent of cows were in their fourth or greater lactation. Average days in milk (DIM) was 159 days with 21.8% of cows in the first 60 DIM and 15% beyond 250 DIM. For analysis, DIM was categorized as early (<31), mid (31–75), late (76–280), and extended (>280) stage of lactation.

### Sequence Data Processing

The single run of MiSeq yielded 2,413,651 250 bp paired-end reads. The library size for the samples varied from 5,474 to 60,280 with a median library size of 18,254 reads. After the quality trimming and initial filtering, the library size varied from 4,269 to 44,535 reads, with a median library size of 13,777 reads and an average library size of 15,436 reads. The samples were not rarified for analyses, as this has been questioned by McMurdie and Holmes (2014). The median read length of quality checked and merged reads was 302 bp. The *de novo* clustering in QIIME yielded a total of 60,655 OTUs of which 22,495 OTUs had less than four reads and were thus removed. Of the ten negative control samples, three contained reads, ranging in number from 3 to 378 that were assigned to OTUs found in all samples not just their respective control groups. This indicates possible erroneous assignment of reads to control samples or minor contamination (cross-talk). Therefore, control samples were removed for the downstream analyses. Additionally, 32,807 OTUs were found to contain less than three reads in 2% of the samples (*n* = 3) and were thus removed. The resulting final OTU table had 5,353 OTUs across 150 samples. Sequencing depth calculated as Zhang-Huang’s metric was 100% after filtering out OTUs found below a mean relative abundance of 0.0005% from original table of 60,655 OTUs.

### Factors Affecting the Diversity of the Microbiota

**FIGURE 1:**
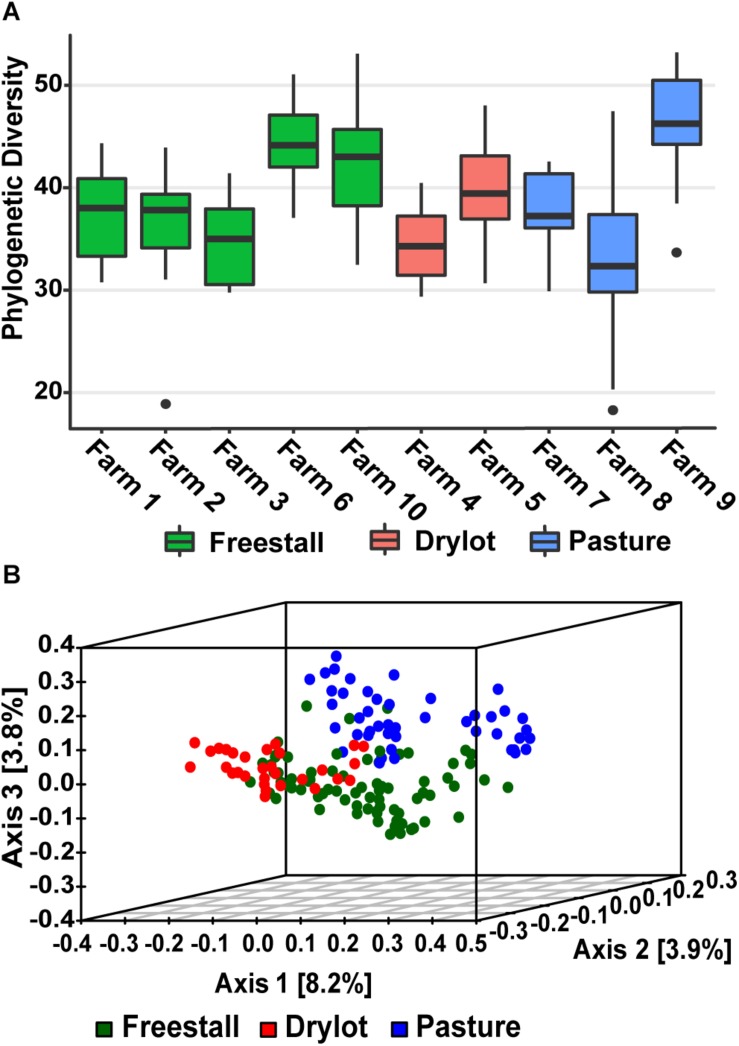
Alpha and beta diversity of samples. **(A)** Average Phylogenetic Diversity (PD) across farms was determined by averaging the sum of all branch lengths of the OTUs present in each fecal sample (Faith’s PD). Alpha diversity of fecal bacterial communities was not significantly affected by housing type, but farm did significantly (*p* = 4.9 × 10^–10^) affect alpha diversity. **(B)** Beta diversity of samples graphed as PCoA of unweighted UniFrac distances colored by housing type. Pasture farms cluster way from the other housing types and are more similar to freestall then dry lot farms.

Alpha diversity calculated as Faith’s PD – the sum of all the branch lengths from the phylogenetic tree generated from the OTUs present in a sample. Alpha diversity was not significantly different across housing type while individual farms did have a significant effect on the alpha diversity (*p* = 4.9 × 10^–10^) (Figure 1A). In addition, production parameters including stage of lactation, parity and age did not have significant effect on the alpha diversity, but the type of forage (*p* = 0.0014), silage (*p* = 0.0048), grains (*p* = 8.41 × 10^–5^), by-products (*p* = 0.0059) and concentrates (*p* = 0.0097) fed did. When the samples were compared to each other using unweighted UniFrac distances, the PCoA ordination revealed that the samples from the pasture farms clustered away from the samples from other housing types (Figure 1B). Within pasture farms, there were two distinct clusters. The first cluster contained a pasture-only farm, farm 9, and the second cluster had two pasture-based farms (farms 7 and 8) that supplemented the cattle’s diet with grain (Supplementary Figure S1). Samples from drylot and freestall cattle also clustered with their respective housing type with a minor overlap (Figure 1B). Some farms showed wide variability across samples, for example farm 8, while samples from other farms such as farm 9 and farm 6 clustered tightly (Supplementary Figure S1). This clustering pattern based on housing type or farm was statistically significant as determined by PERMANOVA (*p* = 0.001). Farm was a stronger driver of the observed ordination pattern as it described 21% of the variation, while housing type only accounted for 6% of the variation. This clustering pattern illustrates the bacterial community structure varies among individual farms with housing type also playing a role.

### Taxonomic Composition of the Community

**FIGURE 2:**
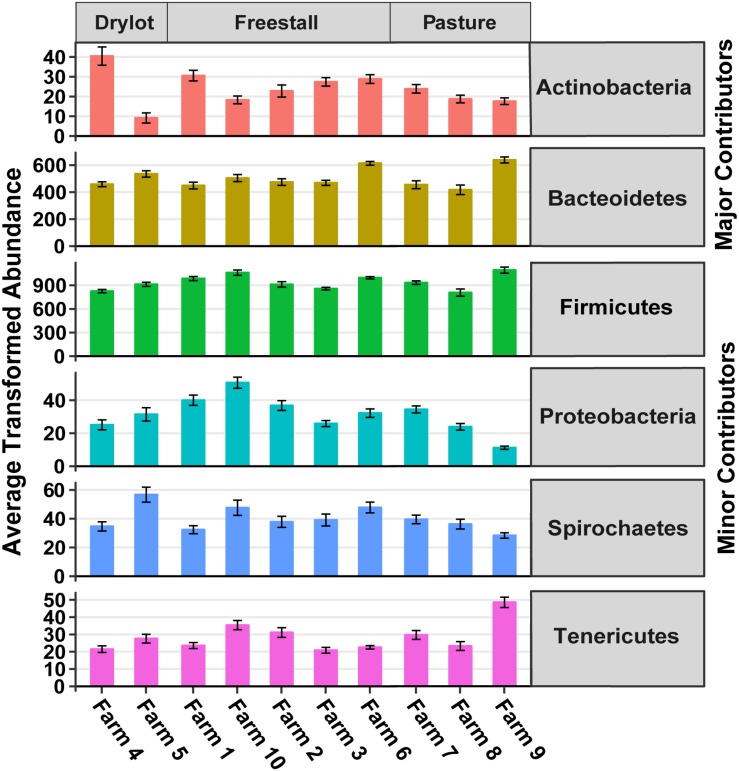
Transformed average abundance ± SEM of major and minor contributing phyla across farms. Abundance of OTUs were transformed with the R package DESeq2 to account for differences in library sizes. Farm significantly affected transformed abundance of all phyla (*p* < 0.001). Housing type affected transformed abundance of all major and minor contributing phyla except Spirochaetes and Bacteroidetes (*p* ≤ 0.01). Determined by Kruskal–Wallis rank sum test.

There were 13 phyla observed in the samples. Firmicutes and Bacteroidetes were the dominant phyla across all farms and together made up approximately 91% of the community, hence these were considered the major contributing phyla (Table 1). Four other phyla, Spirochaetes, Proteobacteria, Tenericutes, and Actinobacteria, accounted for another ∼8% of the community and thus were termed minor contributing phyla (Table 1). The abundance of all phyla varied significantly among individual farms (*p* < 0.001). However, housing type affected abundance of all minor and major contributing phyla except Spirochaetes and Bacteroidetes, as determined by Kruskal–Wallis rank sum test (*p* ≤ 0.01) (Figure 2). Additionally, Verrucomicrobia, Cyanobacteria, Fibrobacteres, Lentisphaerae, Planctomycetes, and Elusimicrobia were present at a relative abundance of less than 0.25% each (Table 1). One archaeal phylum, Euryarchaeota, was observed at a low (0.21%) relative abundance.

**TABLE 1:** Phyla present across farms.

*Phyla*	Average relative abundance (%)^a^
*Firmicutes*	$59.74\pm 3.58^{*}$
*Bacteroidetes*	31.54±3.99
*Spirochaetes*	2.554±0.97
*Proteobacteria*	$2.02\pm 0.94^{*}$
*Tenericutes*	$1.77\pm 0.58^{*}$
*Actinobacteria*	$1.56\pm 0.92^{*}$
*Verrucomicrobia*	$0.24\pm 0.25^{*}$
*Cyanobacteria*	$0.23\pm 0.28^{*}$
*Euryarchaeota*	$0.21\pm 0.13^{*}$
*Fibrobacteres*	$0.08\pm 0.12^{*}$
*Lentisphaerae*	$0.02\pm 0.04^{*}$
*Planctomycetes*	0.02±0.03
*Elusimicrobia*	0.01±0.03

^a^± standard deviation of phyla present in filtered data.

^*^Housing type significantly affected average transformed relative abundance of phyla as determined by Kruskal–Wallis rank sum test (p ≤ 0.01).

**FIGURE 3:**
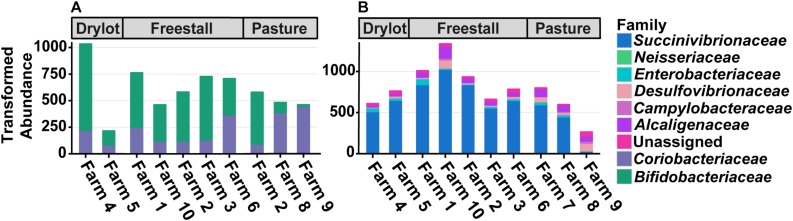
Differences in total transformed abundance of families from **(A)** Actinobacteria and **(B)** Proteobacteria. Two out of three pasture-based farms had a decreased abundance of *Bifidobacteriaceae* compared to *Coriobacteriaceae* while the other had the reverse, mirroring many of the other farms. Both housing type and farm significantly affect the ratio of the two families (*p* ≤ 0.05). Farm 9 that was strictly pasture-based had the lowest abundance of *Succinivibrionaceae* and the highest amount of *Campylobacteraceae* and *Desulfovibrionaceae*. These trends are not seen on Farm 7 and 8, which are pasture-based as well, but did receive grain supplementation.

Five OTUs assigned to genera with pathogenic members that are zoonotic and have been found in cattle were present in a subset of samples. Two OTUs assigned to the genus *Campylobacter* were found in 28 and 12% of samples. In addition, there were three OTUs assigned to the family *Enterobacteriaceae* that were found in 48, 38, and 8% of samples. Despite being found in many samples the abundance of these OTUs was relatively low with the highest average relative abundance of *Enterobacteriaceae* and *Campylobacter* on a farm being 0.089 and 0.030%, respectively. Total abundance across farms is presented in Figure 3B. Representative sequences from these OTUs were placed on a reference tree of full length 16S ribosomal RNA gene sequences from *Escherichia coli* strains, relevant *Salmonella* serovars and *Campylobacter* species. None of the reads originally assigned to *Enterobacteriaceae* were placed within the *Salmonella* clade, but rather these query sequences shared 99% sequence identity with *E. coli* reference sequences and were placed within this monophyletic group in the tree. Better resolution of OTUs originally assigned to *Campylobacter* was not achieved.

### Shared OTUs and Core Fecal Microbiome

Only one OTU, assigned to the family *Ruminococcaceae* was shared across all samples. Fifteen OTUs were found in 95%

of the samples, which made up a total relative abundance of 12.94 ± 3.33% across samples (Table 2). Two OTUs from *Ruminococcaceae* were common in all samples from freestalls and drylots. Pasture farms shared three OTUs with freestall farms compared to having only one in common with drylot farms. At a taxonomic level, families shared across all samples were *Peptostreptococcaceae*, *Ruminococcaceae*, *Rikenellaceae*, *Prevotellaceae*, *Paraprevotellaceae*, *Porphyromonadaceae*, *S24-7*, *Bacteroidaceae*, *Spirochaetaceae*, *Coriobacteriaceae*, *Turicibacteraceae*, *Clostridiaceae*, *Mogibacteriaceae*, *Lachnospiraceae*, and *Erysipelotrichaceae.* In addition, all samples shared three unassigned families belonging to orders Bacteroidales, RF39 and Clostridiales.

**TABLE 2:** Core fecal microbiota of commercial dairy cattle in California.

Phylum	Class	Order	Family	Genus^a^
Actinobacteria	Coriobacteriia	Coriobacteriales	*Coriobacteriaceae*	Unassigned
Bacteroidetes	Bacteroidia	Bacteroidales	*Rikenellaceae*^b^	Unassigned
			*Prevotellaceae*	*Prevotella*
			*Paraprevotellaceae*	*CF231*
				*YRC22*
			*S24-7*	Unassigned
			*Porphyromonadaceae*	*Parabacteroides*
			Unassigned	Unassigned
			*Bacteroidaceae*^b^	5-7N15
				Unassigned
Firmicutes	Clostridia	Clostridiales	*Peptostreptococcaceae*^b^	Unassigned
			*Ruminococcaceae*^b^	*Oscillospira*
				*Ruminococcus*
				Unassigned^c^
			*Clostridiaceae*^b^	*Clostridium*
				Unassigned
			Unassigned	Unassigned
			*Mogibacteriaceae*	*Mogibacterium*
				Unassigned
			*Lachnospiraceae*^b^	*Coprococcus*
				*Butyrivibrio*
				*Dorea*
				Unassigned
	Erysipelotrichi	Erysipelotrichales	*Erysipelotrichaceae*	Unassigned
	Bacilli	Turicibacterales	*Turicibacteraceae*^b^	Turicibacter
Tenericutes	Mollicutes	RF39	Unassigned	Unassigned
Spirochaetes	Spirochaetes	*Spirochaetales*	*Spirochaetaceae*	*Treponema*

*^a^Shared Genera found in 95% of samples.*

*^b^OTU belonging to these families found in 95% of samples.*

*^c^OTU belonging to this genus found in all samples*.

At the genus level, 27 genera were shared among all samples (Table 2). The genera found in all samples of a housing type were defined as the core genera of the respective housing type. Some genera included in the core of a housing type can be found in samples from another housing type. Genera unique to freestall farms were *Anaerovibrio*, *Succinivibrio*, *Roseburia* and an unassigned genus from the family *Succinivibrionaceae*. Genera *Paludibacter*, *Methanobrevibacter*, SMB53 and *Eubacterium* were the core of drylot farms. The only genus unique to pasture farms was *Sutterella*. As with OTUs, samples from pasture farms had more genera in common with freestalls than drylots.

### Effect of Housing Types on Community Composition

**FIGURE 4:**
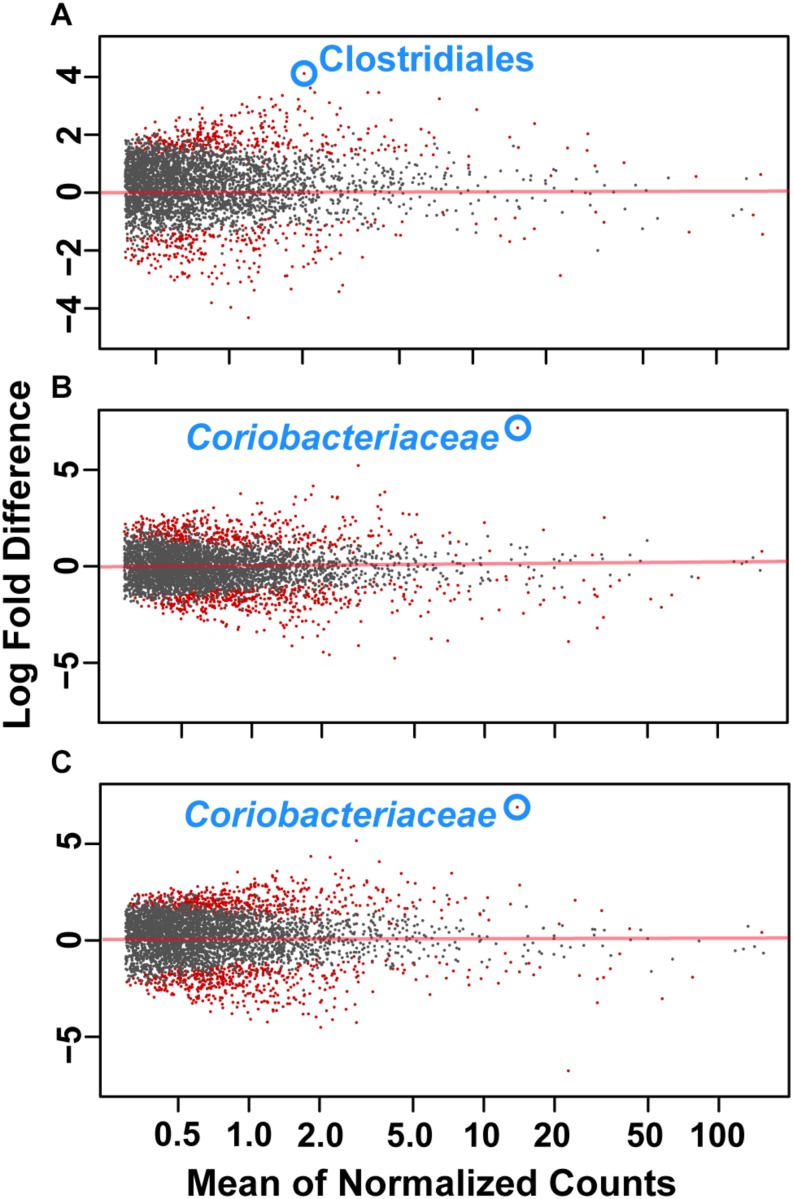
Log2 fold-difference in OTU abundance from samples comparing **(A)** freestall to drylot **(B)** pasture to freestall and **(C)** pasture to drylot housing. Significantly changed OTU abundance are shown in red determined by an adjusted *p* ≤ 0.01. The most significantly changed OTU is circled and labeled with assigned taxonomy. The family *Coriobacteriaceae* showed the most significant change on pasture farms compared to both non-pasture systems while the order Clostridiales was most significantly changed in freestall farms compared to drylots.

**FIGURE 5:**
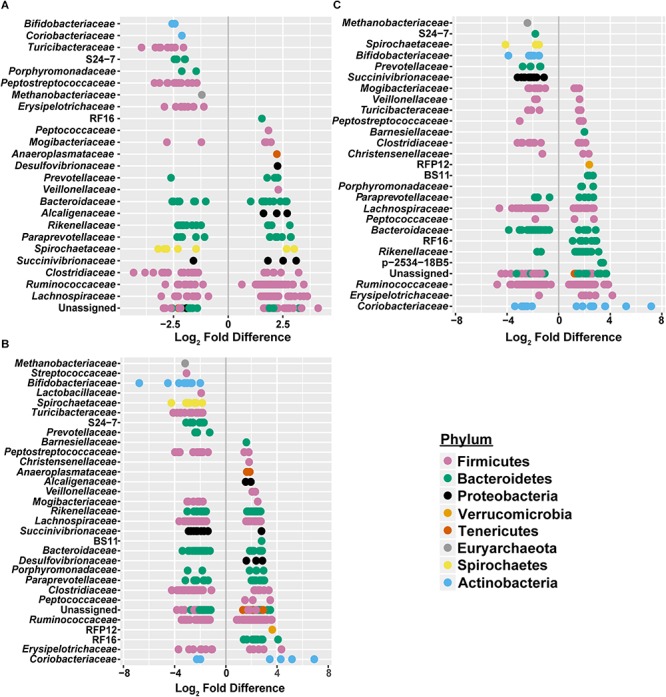
Significant log2 fold-differences of bacterial families’ abundance between **(A)** freestall and drylots **(B)** pasture and freestalls and **(C)** pasture and drylots are presented. Each point is colored by its assigned phyla and represents an OTU that showed a significant difference in abundance between the two housing types compared. Negative values denote a log2 fold reduction of that OTU in the first housing type compared to the second. Similarly, positive values denote log2 fold increases of an OTU in the first housing type compared to the second. Comparisons of between housing types shared log2 fold differences in many of the same families. Adjusted *p* ≤ 0.01 considered significant.

To assess how samples differed from one another, a broad approach was taken and differences were evaluated based on housing type. There were significant differences observed in OTU abundances across housing types. When samples from drylot farms were compared to those from freestall farms, 365 OTUs were present in higher abundance in drylot farms and 267 OTUs present in higher abundance in the freestall farms (adj *p* ≤ 0.05) (Figure 4A). Pasture farms had a higher abundance of 438 OTUs and lower abundance 506 OTUs compared to those in freestalls (adj *p* ≤ 0.05) (Figure 4B). Lastly, samples from pasture farms had 531 OTUs with higher abundance and 500 OTUs with lower abundance when compared to drylot samples (adj *p* ≤ 0.05) (Figure 4C). When comparing pasture to other housing types, the most significant differentially abundant OTU belonged to the family *Coriobacteriaceae*, while for the freestall to drylot comparison, it belonged to the order Clostridiales (Figure 4). For these comparisons, all the OTUs differentially abundant at an adjusted *p-*value ≤ 0.01 are depicted in Figure 5 grouped by their assigned family (Supplementary Table S3). Within the phylum Actinobacteria, the ratio of abundance of OTUs belonging to *Bifidobacteriaceae* and *Coriobacteriaceae* was significantly different among the housing types (*p* = 1.1 × 10^–5^) and farm (*p* = 8.6 × 10^–14^) with pasture-based farms having significantly higher abundance of *Coriobacteriaceae* and a lower abundance of *Bifidobacteriaceae* (Figure 3A). Additionally, pasture farms had a significantly lower abundance of *Succinivibrionaceae* compared to drylot and freestall farms (Figure 3B). Farm 9, a solely pasture-based farm, had the highest abundance of *Campylobacteraceae* and *Desulfovibrionaceae* and strikingly low *Succinivibrionaceae* when compared to all other farms. An OTU assigned to *Methanobrevibacter* was present at significantly lower abundance on pasture farms compared to drylot and freestall farms (Figures 5B,C).

### Effects of Diet and Cow Characteristics on the Fecal Microbiome

**FIGURE 6:**
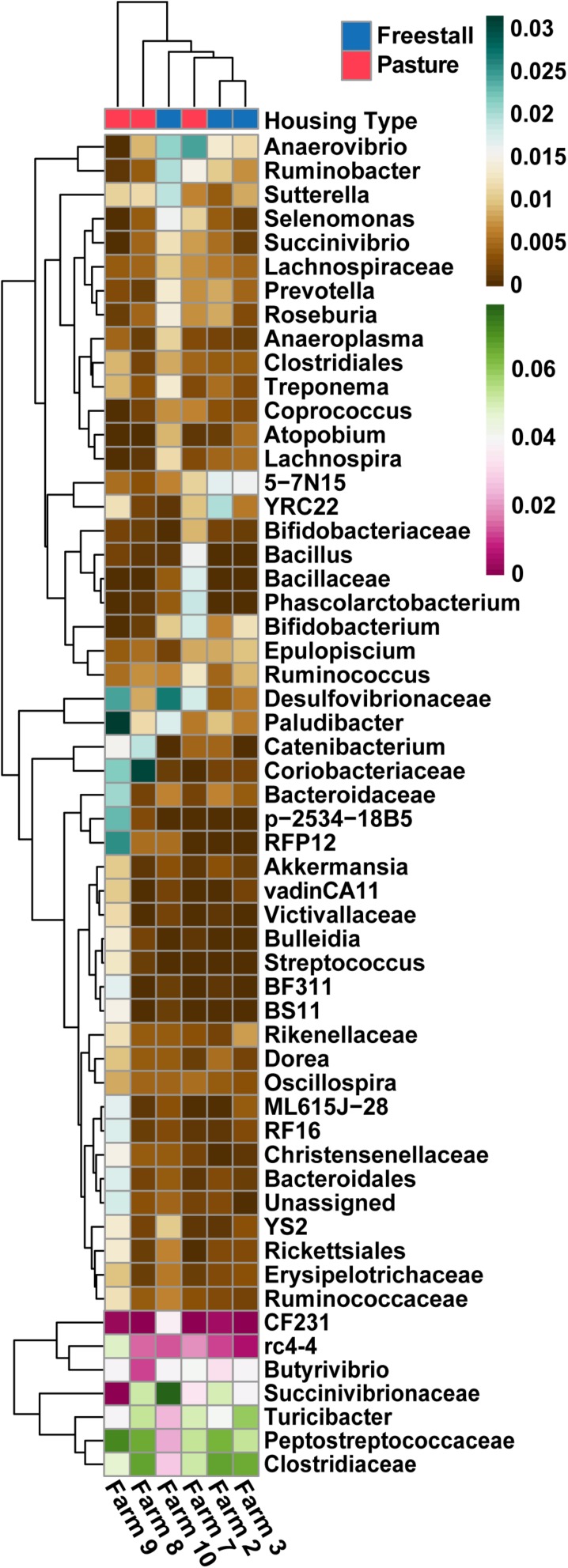
Specific OTUs significantly association with farms (*q* > 0.01), as determined by MaAsLin using a subset of farms with complete metadata, are presented. OTUs were collapsed and combined by their lowest assigned taxonomy and plotted by transformed [arcsin(sqrt)] abundance. Hierarchical clustering shows pasture farms are more similar to each other than freestall farms, with the exception of farm 7. Farm 9, an exclusively pasture farm, shows distinct differences from all other farms and has the highest abundance of *Coriobacteriaceae*.

**FIGURE 7:**
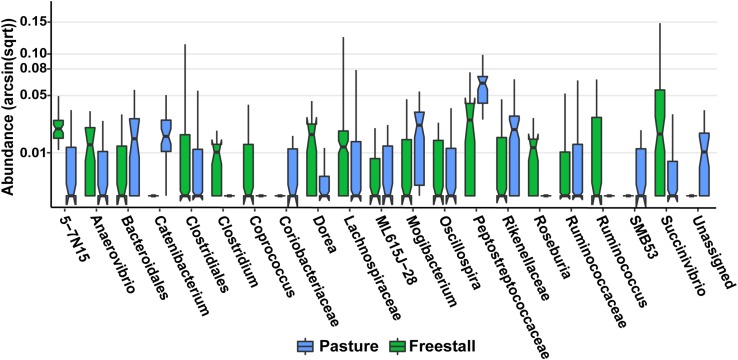
Specific OTUs significantly associated with pasture based farms (*q* > 0.01), as determined by MaAsLin using a subset of six farms from the original dataset that had complete metadata records, are displayed. OTUs were collapsed and combined by their lowest assigned taxonomy and plotted by transformed [arcsin(sqrt)] abundance. Notched box plots show individual arcsine square-root transformed relative abundance’s median as well as the first and third quartiles with notches representing a 95% confidence interval of the median. Pasture farms had significantly higher amounts of *Coriobacteriaceae* even when dietary components, age and stage of lactation were controlled for.

To determine the effects of dietary components and animal characteristics on the microbiota, a subset of six farms, evenly split between freestall and pasture, with complete metadata records were analyzed. When controlling for other factors, the relative abundance of 739 OTUs were significantly associated with farm (Supplementary Table S4). In total, farm 9 had the most significantly associated OTUs (*n* = 241). Notably, OTUs belonging to *Coriobacteriaceae* were overall positively associated (*q* > 0.016) with both farms 8 and 9 while OTUs assigned to *Bifidobacterium* had a negative association (*q* > 0.032) with these farms (Figure 6). Also, a methanogen, *Methanobrevibacter*, was positively associated with farm 7 (*q* > 0.015). The pasture housing type had 88 significantly associated OTUs with *Coriobacteriaceae* remaining positively associated (*q* > 3.0 × 10^–4^) when controlling for differences in diet and animal (Figure 7). Surprisingly, grain was the only dietary variable with significant microbial associations (*n* = 1). An OTU assigned to the family *Bacteroidaceae* and genus 5-7N15 was positively associated with a corn-free diet. In addition, we observed significant associations of specific microbial taxa with parity (*n* = 2), stage of lactation (*n* = 3) and age (*n* = 1).

## Discussion

In this study, we found that the housing type of dairy cattle significantly contributes to the variation in the fecal microbiome of lactating cows. Housing type was significantly correlated to the relative abundances of most major and minor contributing phyla (Table 1 and Figure 2). Clustering based on unweighted UniFrac distances revealed that samples from pasture farms clustered away from samples from other housing types (Figure 1B). Additionally, pasture samples had more OTUs in common with freestall than drylot samples. Collectively, our data demonstrates that samples from freestall and drylot were the most similar to one another and samples from pasture farms were closer to those from freestall farms than drylot farms. Importantly, some OTUs found to be differentially abundant across housing type were also significantly associated with housing type when controlling for dietary components, age and production stages corroborating to the strength of this relationship (Figure 7).

Recently it was shown that shared environments have a greater impact on shaping the microbiota of individuals than host genetics (Rothschild et al., 2018). Thus, the similarity of samples from freestall and drylot is understandable as they share some components of housing and waste management. These shared components include regular scraping or flushing of concrete lanes with reused water from liquid manure storage ponds, use of high-powered fans to circulate air and sprinkler/soaker systems to cool animals. These features may influence the microbes of the built environment that cattle in barns are regularly exposed to as the microbes found in lagoon water, fresh manure and dry manure differ (Pandey et al., 2018). Freestall and drylot farms differ in that drylot animals are housed on arid earthen lots with shaded structures and dried manure as bedding. As the bedding is exposed to environmental elements this changes the microbial composition of the communities residing there that cattle encounter when resting and ruminating (Wong et al., 2016). These conditions are in stark contrast to that of cattle on pasture farms that are at lower stocking density and spend a majority of their time grazing in more open settings. Pasture animals are thus frequently exposed to the microbial communities of soil and plants rather than those in barns. Future research would benefit from using the framework of microbiology of built environments to study how specific management practices on dairy farms change the microbiome on these farms that may in turn influence the gut microbiota of cattle (Gilbert and Stephens, 2018).

While some differences in the microbiota were associated with particular housing types, individual differences on farms also influenced the fecal bacterial community. Farm significantly impacted phylogenetic diversity and average relative abundance of major and minor contributing phyla (Figure 1). Additionally, farm better explained the clustering pattern based on the unweighted UniFrac distances. In fact, when all other variables were controlled for, farm had the most OTUs significantly associated with it (Figure 6). However, farms belonging to the same housing type were more similar despite variation across farms.

A portion of the variation seen among pasture farms might be due to diet, as some pasture-based farms provided supplemental grain. Notably, cattle from Farm 9 were the only ones exclusively pasture raised. This is reflected in the dramatic reduction in the family *Succinivibrionaceae*, in the samples from farm 9 (Figure 3B). Specifically, two genera in this family, *Succinivibrio* and *Ruminobacter*, have been shown to both present in higher relative abundance when grain and fermentable starches are present in the diet (Tajima et al., 2001; Santos and Thompson, 2014) and had a significant negative association with farm 9, farm 8 and the pasture housing type (Supplementary Table S4). However, these associations were determined when diet was broadly controlled for and freestall farms also had negative associations with OTUs from *Succinivibrionaceae.* Taken together, these observations suggest differences in this family are not entirely driven by diet. In line with this, the absence of corn was the only dietary factor that had a significantly associated OTU. This reflects the strong influence of the variable farm as it encompasses differences in diet, genetics, geography, and management practices. A large body of work points to diet as a major factor that affects to the composition of the microbiota (David et al., 2014); however, TMR diets in commercial settings are heterogeneous and this study was not specifically designed to address the effects of specific feedstuffs on the bovine fecal microbiota. Thus, more controlled studies are needed to disentangle effects of diet on the fecal microbiota in cattle.

Pasture influenced the ratio of two Actinobacteria families, *Coriobacteriaceae* and *Bifidobacteriaceae*. *Coriobacteriaceae* abundance was significantly higher in samples from cattle primarily raised on pasture (Figure 3A). Furthermore, when diet, age and lactation stage were controlled for this trend remained (Figures 6, 7). Our findings agree with a previous study of feces of pasture raised beef cattle that found *Coriobacteriaceae* was the most dominant family in the phylum Actinobacteria (Wong et al., 2016). As these beef cattle are maintained on pasture, these results combined with those from our study suggest that access to pasture has an influence on the ratio of Actinobacteria families, thus suggesting this ratio is a result of pasture style management. We found Farm 7 differs from the other two pasture farms in that cattle were housed in a freestall barn during inclement weather with access to a TMR containing silage, and subsequently had a ratio of these Actinobacteria families similar to freestall and drylot farms. Breed is another important difference between these two farms as samples from farm 7 came from Holsteins while, those on farms 8 and 9 were from Jerseys. While the variation in *Coriobacteriaceae* and *Bifidobacteriaceae* might be explained by breed we are unable to test this hypothesis in this dataset, as each farm only had one breed. While differences have been reported in the rumen microbiota in different breeds of dairy cattle (Paz et al., 2016; Zubiria et al., 2018), to our knowledge a similar evaluation of the fecal microbiota in dairy cows has not been carried out. Alternatively, the significant difference in the ratio of *Coriobacteriaceae* to *Bifidobacteriaceae* we observed between drylot or freestall cows and pasture cows might be a function of another variable present on farms 8 and 9 not measured in this study.

*Coriobacteriaceae* is a relatively recently described family that plays an important role in metabolism of B vitamins, conversion of bile salts and steroids, and activation of dietary polyphenols for their hosts, but a full understanding of the mechanisms involved is an area of ongoing investigation (Hylemon et al., 2006; Clavel et al., 2014; Salem et al., 2014). Some members of *Coriobacteriaceae* can convert dietary phytoestrogens into equol, which is a potent isoflavone metabolite capable of binding to the estrogen receptors (Clavel and Mapesa, 2013). This could potentially have implications for host hormonal homeostasis. Considering poor reproduction is a common cause for culling in dairy cattle (California Department of Food and Agriculture [CDFA], 2014), abundance of *Coriobacteriaceae* could be of great interest to the dairy industry. While the function of *Bifidobacteriaceae* and *Coriobacteriaceae* has not been clearly defined in adult cattle, future metagenomic and transcriptomic studies will help elucidate their metabolic role in the microflora of cattle.

One of the most striking findings in this study was the lack of OTUs shared across all samples. These data suggest a core fecal microbiome defined at the OTU level may not be determined for dairy cattle. A previous survey of fecal microbiota of 30 beef cattle on different feedlot operations found that individual cattle only shared nine OTUs (Shanks et al., 2011). Our data suggest that a larger sample size in that study could eliminate these shared OTUs. Our data are congruent with a 2009 study in 154 people in which there was not a single abundant bacterial species, defined as >0.5% of the community, common among all individuals (Turnbaugh and Gordon, 2009). For this reason, the Human Microbiome Project suggested a core be defined by functional pathways (Bäckhed et al., 2012). Such redundancy in metabolic pathways in the rumen has been proposed, but has not yet been directly determined (Weimer, 2015). These data speak to the enormous species and potentially strain-level diversity shaped by environment and host immune system. Our study included heterogeneous diets, genetics and management practices, potentially reducing the ability to identify shared core OTUs. However, because this study used samples from commercial settings, it presents a more realistic representation of a core microbiota.

In contrast to our data, Kim et al. (2014) reported that 1,286 OTUs were shared across 333 steers. The discrepancy between our and their study is likely due to differences in primers (V1–V3 vs. V4) and the stringent filters in our study to remove sequencing artifacts. While there is no “best” primer set to use, the V1–V3 (27F/28F-518R/519R) primers only amplify 21.6% of known bacterial sequences in the RDP database, potentially skewing the diversity metric (Fang and Zhang, 2015). Despite poor amplification of *Propionibacterium* by our primer pair (V4), it is appropriate for amplifying a diverse community as they target a much larger diversity of bacteria (62.8% of bacterial sequences in the RDP database) (Walters et al., 2011; Fang and Zhang, 2015). The choice of primers impacts results, thus verification of the core microbiota, if it exists, would be best determined by primer-free full length 16S sequencing (Karst et al., 2018) or through shotgun metagenomics.

Hence, in this study, we define the core taxa in feces as the 27 identified genera present in all samples (Table 2). Of the two studies that investigated the fecal microbiota of cattle, one reported 30 taxa present in all samples, but only reported at the level of phylum (Shanks et al., 2011). The other found consistent presence of highly abundant genera – *Clostridium*, *Porphyromonas*, *Bacteroides*, *Ruminococcus*, *Alistipes*, *Lachnospira*, and *Prevotella* – in 20 individuals (Dowd et al., 2008). While the families of all these genera are present in our proposed core, *Bacteroides*, *Alistipes*, and *Porphyromonas* are not part of our definition of core. Also, of the 6 other genera found in the 20 cows in their study, only *Oscillospira* is part of the core that we observed in 150 cows. Together, these observations suggest that there may be a core defined by the highly abundant genera, but the presence of other genera will depend on factors such as environment and host-diet interactions.

While we find a consensus at the genera or family level, due to the diversity of metabolic potential among species, a core defined by taxonomy maybe misleading when making conclusions about functional differences. It is likely that there is redundancy in metabolic function of the hindgut microbiota similar to what was hypothesized about the rumen microbiome (Weimer, 2015). There has been some early evidence supporting this hypothesis, where a convergence of function has been shown in the rumen of two beef steers, despite the taxonomic differences between their microbiota (Taxis et al., 2015). Thus, at this point defining the core microbiota at a phylum level remains too broad to be meaningful. On the other hand, defining it at an OTU level is too narrow to account for metabolic diversity; hence, providing little insight into the microbial metabolic network. However, metagenomic and transcriptomic studies with larger sample sizes are required to confirm the convergence of function hypothesis and to understand the ecophysiology of the microbiome in dairy cattle.

With multi-omic research, such as metagenomics, future studies might be to identify the bacterial species involved in the microbial metabolism in feces, such as nitrogen cycling. Recent work has characterized some genera in the rumen putatively involved in nitrate reduction and denitrification; yet, their metabolic role in the lower digestive tract has yet to be determined (Latham et al., 2016). Although there are tools available to predict the functional capacity from 16S rRNA sequences, like PICRUSt, their accuracy relies on available reference genomes (Langille et al., 2013). Currently, there is at least a 10-fold difference in the number of OTUs present in our data set and the available reference genomes from bovine microbiome, reducing the usefulness and accuracy of such predictive tools.

Similar to functional prediction, precise detection of pathogens was unfeasible with our data. Despite 58 and 31% of cows in this study having at least one OTU assigned to the family *Enterobacteriaceae* and genus *Campylobacter*, respectively, the relative abundance of these organisms is low. Because only 7.6% of samples from dairies in the western United States were positive for *E. coli O157* (Animal and Plant Health Inspection Service, 2003), there may be a commensal population of *Enterobacteriaceae* that is part of the “normal” microbiome of cattle. Our results suggest that OTUs assigned to the family *Enterobacteriaceae* were related to *Escherichia* and not *Salmonella*. This is lower than a survey of California dairy cull cows, that found approximately ∼3.4% of cows had a positive culture for a *Salmonella* serovar (Abu Aboud et al., 2016). Despite the presence of *Escherichia* in our samples, we cannot say conclusively if these are pathogenic or commensal. Thus, PCR and culture-based methods remain the gold standard for routine detection of these pathogens (Clermont et al., 2013).

## Conclusion

The large number of individuals and the robust analysis presented in this survey of fecal microbiota from commercial dairy cows are important refinements to current understanding of the microbial ecology of cattle feces. As we understand more about the effect of the built environment on its occupants and their microbiota this can better inform animal and waste management practices on dairy farms. While this study cannot completely separate effect of diet from farm-to-farm variation, the large data set allows for insights into future areas of study and contributes to the further elucidation of the core microbiota of dairy cattle. We propose that a core microbiota at the genus level is present in the feces of commercial dairy cattle, across a wide array of housing types and diets (Table 2). Additionally, the vast variation at a deeper taxonomic level does not allow for identification of core microbiota at an OTU level. Based on our data, the ratio of *Coriobacteriaceae* and *Bifidobacteriaceae*, two families in phylum Actinobacteria, appear to be related to a pasture-based diet. Further studies using metagenomics are needed to determine the metabolic capability of the fecal microbiota specific to dairy cattle.

## Author Contributions

JVH prepared libraries, analyzed and interpreted data, and wrote manuscript. SB substantially contributed to data analyses and manuscript editing. DM, JMH, and BK identified farms for study, collected samples, and reviewed manuscript. PP compiled metadata, oversaw sample collection, and reviewed manuscript. DM and EM conceived the project, obtained funding, developed experimental design, and edited manuscript.

## Conflict of Interest Statement

The California Dairy Research Foundation (CDRF) was provided with an advance copy of the manuscript prior to publication. No changes were requested or made on behalf of CDRF. The authors declare that the research was conducted in the absence of any commercial or financial relationships that could be construed as a potential conflict of interest.

## Supplementary Material

FIGURE S1 Beta diversity of samples graphed as PCoA of unweighted UniFrac distances colored by Farm. Clustering shows some farms have greater diversity within farm individuals. Farm 9 and Farm 6 clustered tightly while individual samples from Farm 8 showed greater variability. This clustering pattern illustrates the bacterial community structure varies across individual farms to a smaller extent with housing type being the dominant factor.

TABLE S1 Metadata for each sample used for analysis.

TABLE S2 Presence or absence of specific feedstuffs in diet of farms where feed information was collected.

TABLE S3 The full output of differential abundance testing as determined by DESeq2. All differentially abundant OTUs are listed with their assigned taxonomy. Columns are as follows baseMean is the average of the normalized count values, log2FoldDifference is the effect size estimate and lfcSE is the standard error estimate for the log2 fold change estimate. The subsequent columns are the Wald test statistic its *p*-value and adjusted p-value as calculated by the Benjamini-Hochberg (BH) adjustment for multiple tests.

TABLE S4 A subset of farms (2–3, 7–8, 10) that had complete records of metadata were analyze with MaAsLin in R. All OTUs associated with individual metadata as determined are listed with their assigned taxonomy, coefficients, *p* and *q* values.
