# Supplementary figures and images for: Fecal Microbial Communities in a Large Representative Cohort of California Dairy Cows
Source: Front Microbiol. 2019 May 16;10:1093. doi: 10.3389/fmicb.2019.01093 (PMC6532609; doi:10.3389/fmicb.2019.01093)

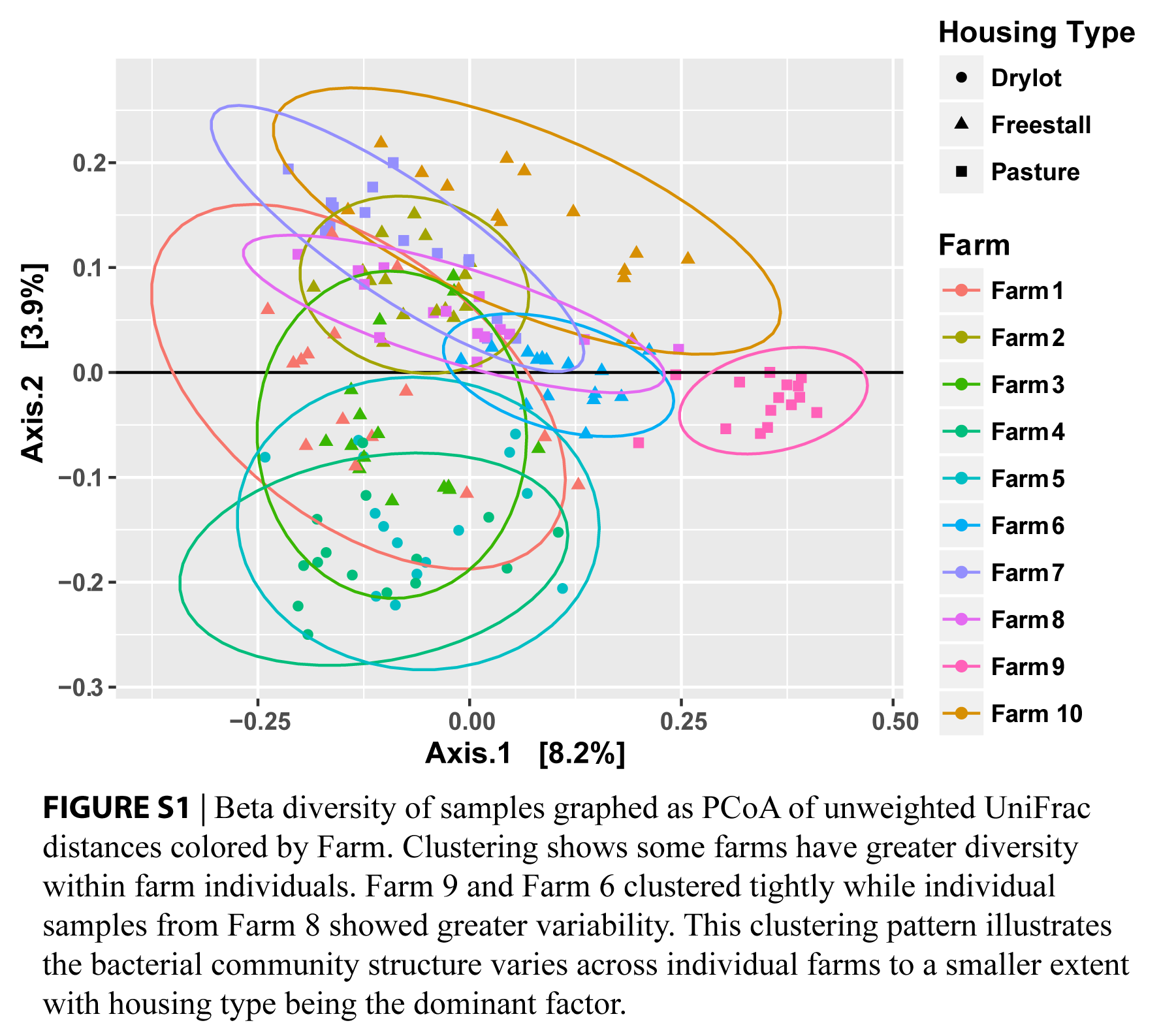

Supplement: FIGURE S1 — Beta diversity of samples graphed as PCoA of unweighted UniFrac distances colored by Farm. Clustering shows some farms have greater diversity within farm individuals. Farm 9 and Farm 6 clustered tightly while individual samples from Farm 8 showed greater variability. This clustering pattern illustrates the bacterial community structure varies across individual farms to a smaller extent with housing type being the dominant factor. [file Image_1.TIF]
